# One‐dimensional carbon based nanoreactor fabrication by electrospinning for sustainable catalysis

**DOI:** 10.1002/EXP.20220164

**Published:** 2023-05-11

**Authors:** Fagui He, Yiyan Wang, Jian Liu, Xiangdong Yao

**Affiliations:** ^1^ State Key Laboratory of Catalysis, Dalian Institute of Chemical Physics Chinese Academy of Sciences Dalian Liaoning China; ^2^ DICP‐Surrey Joint Centre for Future Materials, Department of Chemical and Process Engineering, and Advanced Technology Institute University of Surrey Guilford Surrey UK; ^3^ Shanghai Key Laboratory of Molecular Catalysis and Innovative Materials Fudan University Shanghai P. R. China; ^4^ School of Advanced Energy Sun‐yat Sen University (Shenzhen) Shenzhen Guangdong China; ^5^ State Key Laboratory of Green Chemical Engineering and Industrial Catalysis, Shanghai Research Institute of Petrochemical Technology Sinopec Shanghai China

**Keywords:** 1D fibrous nanostructure, carbon‐based nanoreactors, electrocatalyst, electrospinning, energy storage

## Abstract

An efficient and economical electrocatalyst as kinetic support is key to electrochemical reactions. For this reason, chemists have been working to investigate the basic changing of chemical principles when the system is confined in limited space with nanometer‐scale dimensions or sub‐microliter volumes. Inspired by biological research, the design and construction of a closed reaction environment, namely the reactor, has attracted more and more interest in chemistry, biology, and materials science. In particular, nanoreactors became a high‐profile rising star and different types of nanoreactors have been fabricated. Compared with the traditional particle nanoreactor, the one‐dimensional (1D) carbon‐based nanoreactor prepared by the electrospinning process has better electrolyte diffusion, charge transfer capabilities, and outstanding catalytic activity and selectivity than the traditional particle catalyst which has great application potential in various electrochemical catalytic reactions.

## Graphical abstract

Owing to the synergy between highly dispersed active sites and particular substrates with the one‐dimensional nanostructure, fibrous carbon‐based nanoreactors have exhibited fascinating electrocatalytic performance in electrochemical reactions. Therefore, one‐dimensional fibrous carbon‐based nanoreactors from the electrospun fabrication process have been used in catalyzing oxygen reduction reaction, oxygen evolution reaction, and hydrogen evolution reaction electrochemical reactions.

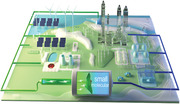

## 1 INTRODUCTION

With the rising issues of energy sources exhaustion and environmental degradation, renewable energy sources acquiring extensive attention while the development of electrochemical energy storage technologies or battery technologies is an effective approach to widely utilizing renewable energy sources. Chemical energy and electrical energy can be directly converted by battery technologies, thus it is particularly suitable for storing electrical energy from all sources. This superiority could help surmount the mismatch between the generation and end‐use which could effectively solve the instability and intermittence of renewable energy sources.^[^ ^1^ ^]^ As a consequence, expediting the development and utilization of sustainable energy sources acquired broader attention in battery technologies.

For fuel cells, metal‐air cells, or electrolytic water hydrogen production technology, developing an efficient and economical electrocatalyst to overcome the reaction energy barrier is the key point to enhancing the reaction rate and reducing the overpotential of the electrochemical reactions. From this perspective, the enzyme around nature is one of the most efficient catalysts as it could catalyze reactions in much more mild and green conditions.^[^ ^2^ ^]^ In the enzyme‐catalyzed reactions, the remarkable efficiency, as well as chemo‐, regio‐, and stereoselectivity has inspired chemists for designing a synthetic system with higher activity and selectivity.^[^ ^3^ ^]^ The micro‐environment within the enzyme cavity can effectively induce important processes such as substrate pre‐organization, protein dynamics, and desolvation of substrates, which significantly affect the enzyme‐catalyzed reaction.^[^ ^4^ ^]^ Therefore, fabricating a binding cavity that is similar to the microenvironment became a key element in constructing artificial enzymes.^[^ ^2^, ^3^ ^]^ Chemists, on the other hand, have struggled to explore the changes in fundamental chemistry when the reaction systems are confined to nanoscale dimensions or sub‐microliter volumes.^[^ ^5^ ^]^ Thus, the accurate design and construction of a confined reaction environment called the reactor, has received increasing attention in chemical, biological, and materials science.^[^ ^6^ ^]^ Nanoreactor, a special type of reactor at the nanoscale, possesses a large portion of recent scientific research, and various types of nanoreactors have been designed and constructed such as nanopores, nanoholes, hollow nanoparticles, porous nanostructures, and tubular nanostructures. With the advanced nanostructures, the nanoreactor system enables the number of atoms or molecules understudied to be tuned and controlled through assorted avenues in ways which is impossible with bulk systems. During the process of chemical reactions, nanoreactors could change the basic chemical nature of molecules and moieties inside as well as alter their behavior. Among all these nanoreactors, nanofibers aroused increasing interest in recent decades due to their attractive properties such as tunable fiber diameter, high aspect ratio, and high surface‐volume ratio.^[^ ^6^, ^7^ ^]^

On the other hand, one‐dimensional nanostructured catalysts such as nanofiber, nanowire, and nanorod show better electrolyte diffusion and charge transfer capability and excellent performance of catalytic activity and selectivity than those of the traditional granular catalysts.^[^ ^8^ ^]^ The advantages of 1D nanomaterial include, but not limited to: (1) sufficient anchoring sites can be introduced by facilely regulating the surface physicochemical properties of 1D nanostructures through various treatment approaches which enables a strong interaction between metal and support; (2) 1D nanostructure could provide large specific surface area which benefits hosting atomic‐level active sites and mass transport; (3) through shortening electron transfer distance, the advanced 1D nanostructure is more favorable for the highly efficient reaction kinetics (4) 1D nanomaterials possess superior flexibility in element selection which enables facilely coupling between 1D nanostructure with other desired nanomaterials to fabricate the catalysts with enhanced catalytic performance; (5) more importantly, carbon‐based 1D nanomaterials also possesses the merits of traditional carbon nanomaterials, such as resistance to acid and alkali media, high‐temperature stability, low cost, and easy recyclability. One‐dimensional fibrous nanomaterials can interweave to form a 3D framework with multiscale porosity for further improving the electrocatalytic performance.^[^ ^9^ ^]^ In addition, the superior flexibility of 1D fibrous nanomaterials can be also used as catalyst supports for providing a possible approach for the construction of the desirable catalysts with an advanced structure. These 1D nanomaterials can be prepared by self‐assembly,^[^ ^10^ ^]^ hydrothermal synthesis,^[^ ^11^ ^]^ template method,^[^ ^12^ ^]^ gas‐phase method^[^ ^13^ ^]^ and electrospinning.^[^ ^14^ ^]^

**SCHEME 1:**
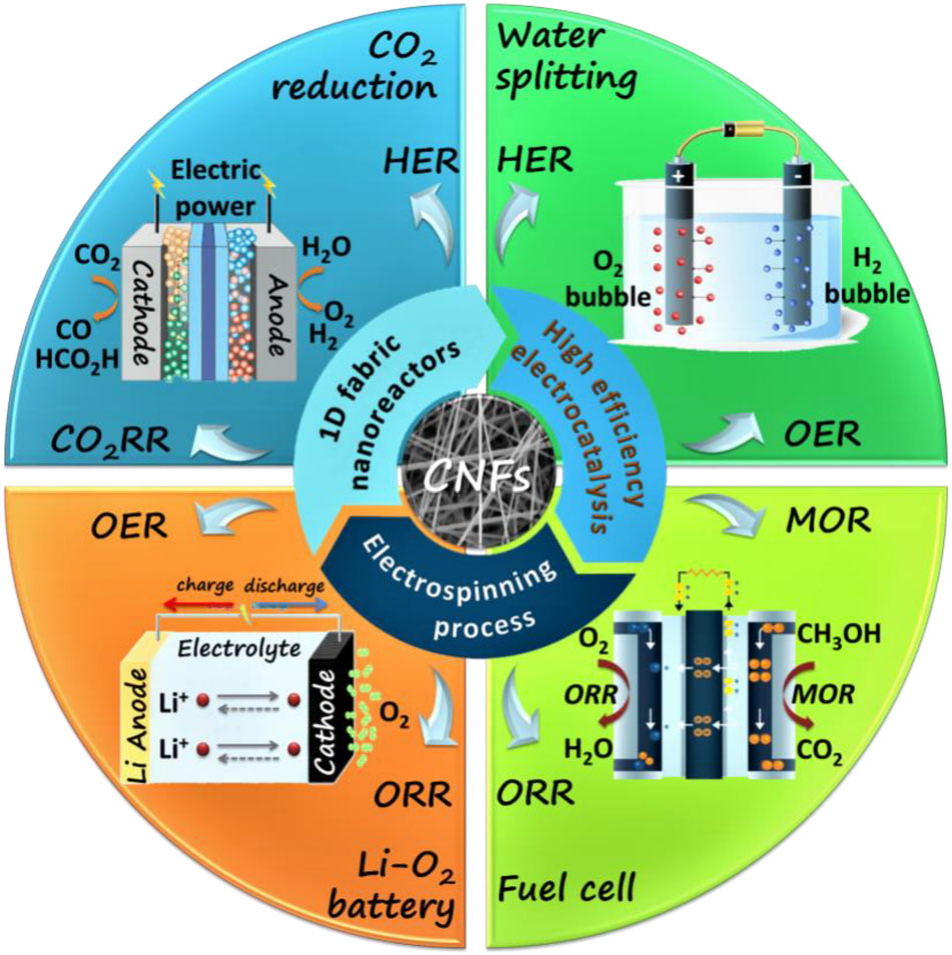
The schematic illustration of the application of CNF catalysts prepared via electrospinning.

Thus, it can be seen that a nanoreactor with 1D fibrous nanostructure will have great potential when used to fabricate high‐efficiency catalysts for various electrochemical reactions. Among various chemical or physical synthetic approaches for generating 1D fibrous nanostructure, electrospinning is a simple, versatile, and low‐cost technique for producing continuous fibrous materials from polymer solutions or melts.^[^ ^15^ ^]^ This approach has been known since it is first patented in the US in 1902.^[^ ^16^ ^]^ Currently, electrospinning became a widely used technique for fabricating continuous fibers with nanoscale diameters. The carbon nanofiber (CNF) catalysts fabricated via electrospinning exhibit superior catalytic activity and electrochemical durability in several applications such as oxygen reduction reaction (ORR), oxygen evolution reaction (OER), hydrogen evolution reactions (HER), methanol oxidation reaction (MOR) and the electrochemical carbon dioxide reduction reaction (Scheme 1).^[^ ^17^ ^]^

With the continuous improvement of the electrospinning technique, CNFs with advanced architectures, such as core–shell nanofibers, porous nanofibers, and hollow nanofibers can be constructed. Meanwhile, the arrangement of nanofibers has become more controllable, from single fibers to ordered fibers.^[^ ^18^ ^]^ This article focuses on the unique features of electrospinning, a widely used technique for fabricating 1D nanofibers with particular morphology, in preparing 1D carbon‐based fibrous nanoreactors for sustainable catalysis. So far, with a surge of interest in the application of electrospun technology, the investigation of this technology is no longer limited to the laboratories but also step wisely moving toward the industry for various applications of nanofibrous materials with ultra‐small diameters.

## 2 BASIC ELECTROSPINNING PROCESS AND PARAMETERS

**SCHEME 2:**
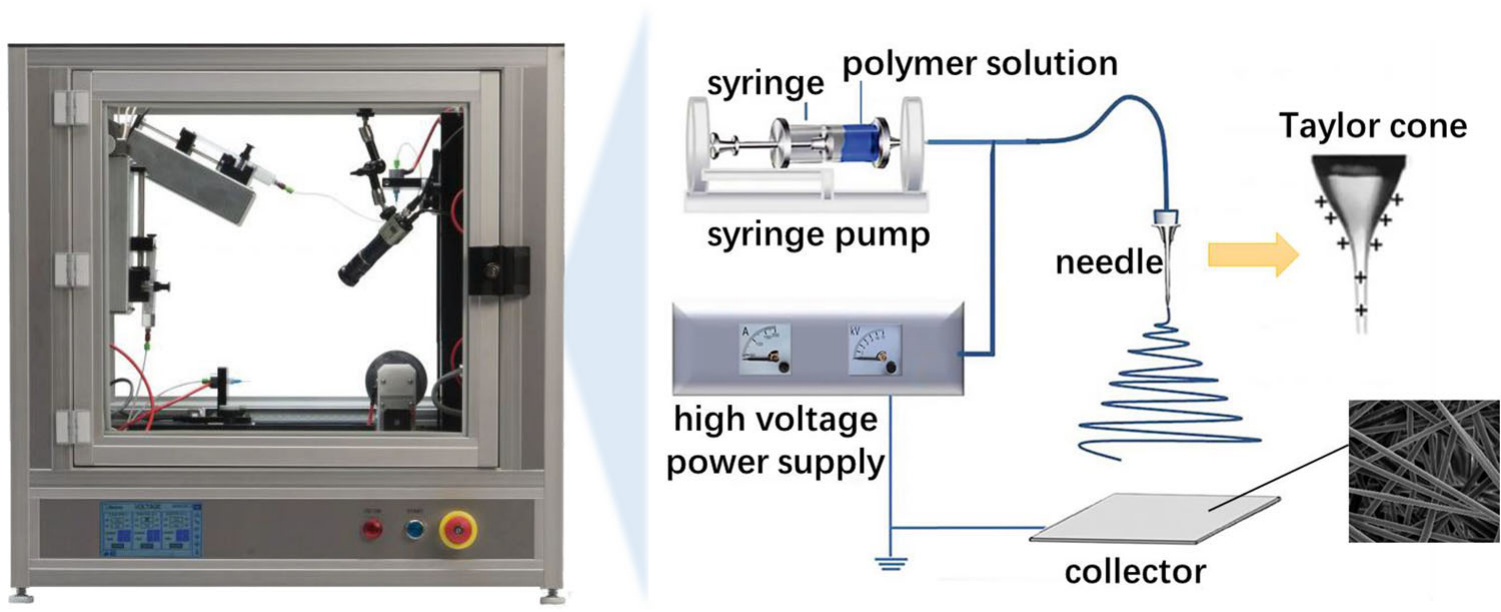
Schematic illustration of the basic setup for electrospinning.

Electrospinning technology was first proposed by Taylor,^[^ ^19^ ^]^ and has been widely used in energy storage system applications. For electrospinning, the key consist requires a high voltage power supply (HVPS), a syringe with a needle (or capillary tube of small diameter) as well as a metal collector.^[^ ^20^ ^]^ As is shown in Scheme 2, the needle is connected to the syringe pump and driven by the syringe pump. After loading an appropriate volume of precursor solution into the syringe, when a certain volume of electrically conductive solution is exposed to an electric field, its shape changes due to surface tension. With increasing the voltage, the influence of the electric field becomes more pronounced, and a Taylor cone forms when the electric field reaches a critical value. Then, the tip of the Taylor cone ejects a charged jet of solution which is the beginning of the electrospinning process.^[^ ^21^ ^]^ The jet will be elongated by an unstable and rapid whipping process between the tip and the collector, meanwhile, the evaporation of the solvent leads to the formation of solidified continuous, uniform fibers with nanometer‐scale diameters.^[^ ^19^, ^22^ ^]^

**SCHEME 3:**
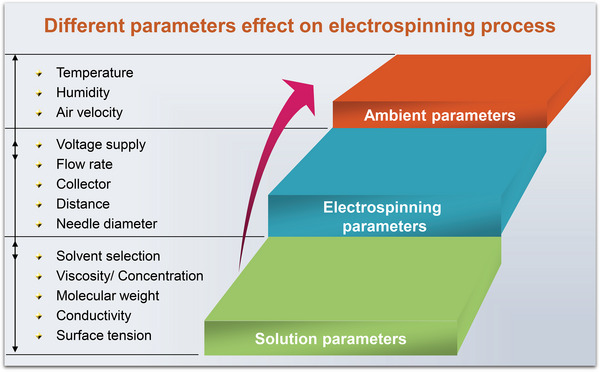
The schematic illustration of the parameters influences the electrospinning process.

In the electrospinning procedure, adjusting the following parameters can realize the controllability of the morphology and diameter of electrospun nanofibers (Scheme 3):^[^ ^16^, ^23^ ^]^

- The molecular weight and its distribution; and the architecture (such as branched and linear) of the polymer;
- Some important properties of the solution (e.g., the surface tension, viscosity, and conductivity);
- Important parameters in the electrospinning process such as the concentration, flow rate, and electric potential;
- Distance between the capillary and collection screen;
- Some ambient parameters (such as humidity and temperature in the chamber);
- Motion of collector;
- The gauge of needle.

In general, the ideal electrospun nanofibers (NFs) would be in that: (1) the diameters and morphology of the nanofiber are homogenous and controllable, (2) defect‐free or defect‐controllable on the surface of the fiber, and (3) the continuous single nanofibers can be collected.^[^ ^23a^ ^]^ However, from the current research results, it is tough for meeting all these requirements at the same time. For example, the diameters of electrospun fibers primarily depend on the jet sizes and the polymer contents in the jets.^[^ ^24^ ^]^ Generally, the fiber diameter mainly depends on the solution viscosity.^[^ ^25^ ^]^ After the solid polymer materials are dissolved in a solvent, the solution viscosity is proportional to the polymer concentration, therefore, the higher concentration of the polymer results in nanofibers with larger diameters.^[^ ^24^, ^26^ ^]^

## 3 APPLICATION OF ELECTROSPINNING IN ELECTROCATALYSTS MATERIALS

**SCHEME 4:**
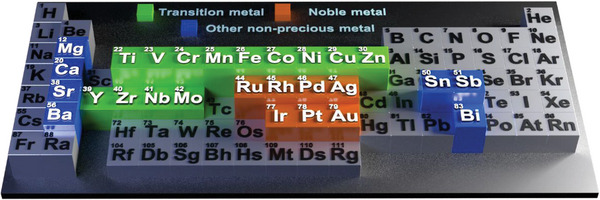
Metal elements that have been widely used for fabricating metal‐based CNFs catalysts.

The electrochemical OER, ORR, and HER are important reactions in renewable energy technologies while the catalysts with high selectivity and reactivity are the key point for these electrochemical reactions.^[^ ^27^ ^]^ As shown in Scheme 4, noble metals such as ruthenium (Ru), palladium (Pd), and platinum (Pt)‐based materials have been widely used for ORR, OER, HER, and MOR reactions.^[^ ^28^ ^]^ However, in consideration of the scarcity (less than 3 wt% among all elements on earth)^[^ ^27f^ ^]^ and the high cost of noble metals, improving the efficiency of noble metal‐based catalysts is of great importance in large‐scale industrial applications. Recently, the utilization of non‐precious metal catalysts (Scheme 4) became a promising alternative, and transition metal‐based catalysts have been developed. Low cost and easy preparation make those catalysts more appropriate for large‐scale applications at the industrial level, which plays a crucial role in the wide utilization of renewable energy sources.

In addition, the electrocatalytic reactions occur at an electrode‐electrolyte interface, and their performance is remarkably affected by the specific surface area and conductivity of the catalyst.^[^ ^29^ ^]^ The one‐dimensional nanomaterials prepared by electrospinning technology are beneficial to reduce the size of the catalyst from micron/submicron to nanometer and directly construct continuous one‐dimensional nanostructures. Meanwhile, the emergence of advanced technologies such as near‐field electrospinning and the development of needleless jets and other devices, opened up the possibility to prepare ordered nanofibers and improve the controllable deposition of electrospun fibers.^[^ ^30^ ^]^ For catalysts prepared by electrospinning technology, controlling the arrangement of fibers can effectively improve the contact range between the catalyst surface and the electrolyte, improve the distribution structure of mesh fibers, promote mass and heat transfer on the catalytic surface, and accelerate the reaction rate. Henceforth, the combination of noble metals and non‐precious metal materials, the doping of non‐metallic materials such as nitrogen, and the design of novel 1D fibrous nanoreactors will significantly improve the catalytic performance of metal‐based CNFs.

### 3.1 Non‐precious metal oxide carbon nanofiber catalysts

Recently, in order to prepare efficient and economical catalysts, more investigations are focused on developing non‐precious metal‐based catalysts.^[^ ^27^, ^31^ ^]^ In addition, the combination between non‐noble metals and a small amount of highly active noble metals (e.g. Pd, Ru, and Pt) can obtain low‐cost catalysts with increased catalytic performance through the synergy between metals.^[^ ^32^ ^]^

#### 3.1.1 Unitary‐ or binary‐transition metal oxide

**FIGURE 1:**
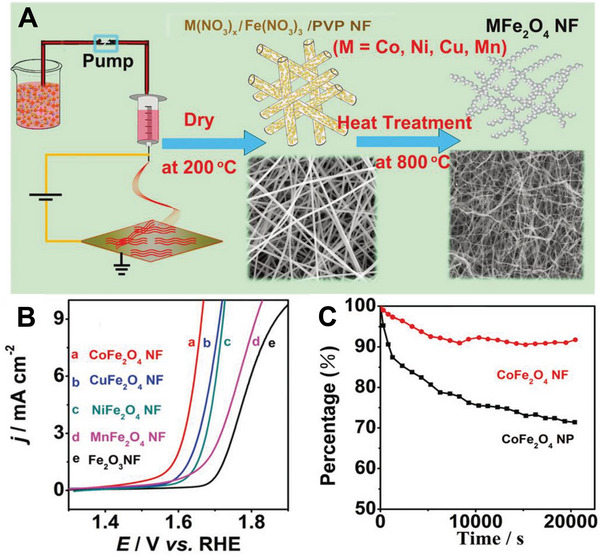
Preparation and electrocatalytic performance of MFe_2_O_4_ NFs. A) The preparation procedures of MFe_2_O_4_ NFs. B) Polarization curves for OER on Fe_2_O_3_, CoFe_2_O_4_, NiFe_2_O_4_, CuFe_2_O_4_, and MnFe_2_O_4_ NFs. C) Chronoamperometric response for the CoFe_2_O_4_ NFs and CoFe_2_O_4_ NPs at 1.8 V vs. RHE. Reproduced with permission.^[^ ^17a^ ^]^ Copyright 2015, Royal Society of Chemistry.

Li et al.^[^ ^17a^ ^]^ synthesized porous transition metal ferrite nanofibers MFe_2_O_4_NFs (M = Co, Ni, Cu, Mn) through electrospinning process (Figure 1A). The abundant micro/meso/macropores are distributed on the surface and within the films of the spinel‐type MFe_2_O_4_ nanofibers. The accessible transport channels provided by electrospun MFe_2_O_4_ nanofibers could successfully reduce the mass transport resistances, resulting in enhanced conductivity and reactivity of the exposed catalytic active sites. The CoFe_2_O_4_ nanofibers exhibit a higher OER activity in 0.1 m KOH electrolyte. The onset potential is 372 mV (Figure 1B) and the value is more negative than other obtained MFe_2_O_4_ NF samples. Remarkably, the CoFe_2_O_4_ NFs possess a current density of 5 mA cm^−2^ at the overpotential of 408 mV.^[^ ^17^, ^33^ ^]^ Additionally, compared with CoFe_2_O_4_ NPs, CoFe_2_O_4_ NFs possess strong durability in alkaline electrolytes. After operation for 20,000 s, CoFe_2_O_4_ NFs show insignificant performance attenuation (< 8%) while CoFe_2_O_4_ NPs demonstrate a rapid activity decrease (> 30%, Figure 1C).

**FIGURE 2:**
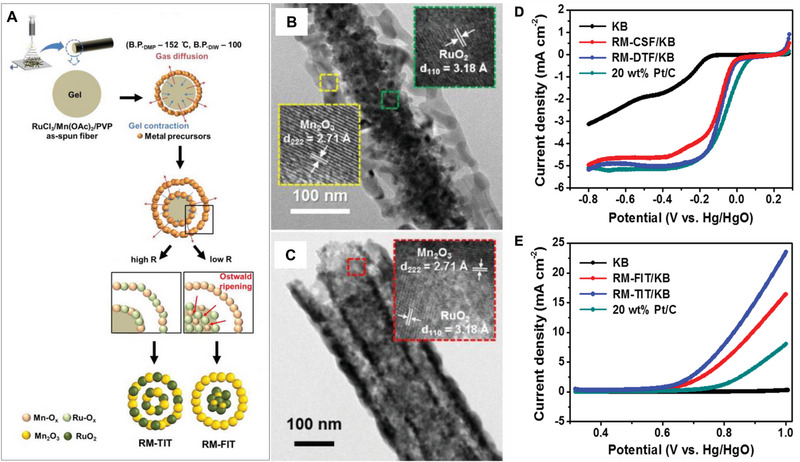
Preparation and electrocatalytic performance of RM‐FIT and RM‐TIT. A) The fabrication procedure of RM‐FIT and RM‐TIT. B) HRTEM image of RM‐FIT. C) HRTEM image of RM‐TIT. LSV curves measured in D) the ORR and E) the OER region of KB, RM‐FIT/KB, RM‐TIT/KB, and 20 wt% Pt/C used air electrodes in 0.1 m KOH (O_2_ saturated). The scan rate is 5 mV s^−1^. The rotation rate for ORR is 1600 rpm. Reproduced with permission.^[^ ^17b^ ^]^ Copyright 2016, American Chemical Society.

In order to improve the conductivity and OER kinetics of MnO*_x_*, Yoon, et al.^[^ ^17b^ ^]^ exploited the phase‐separated RuO_2_/Mn_2_O_3_ fiber‐in‐tube (RM‐FIT) and the multi‐composite RuO_2_/Mn_2_O_3_ tube‐in‐tube (RM‐TIT) (Figure 2). The core fibers (∼80 nm) and shell tubes (∼220 nm) of RuO_2_/Mn_2_O_3_RM‐FIT exhibit distinctive phase separation resulting in cylindrical pores and the two components (Ru and Mn) are distributed in different phases. In contrast, the RM‐TIT has a double‐walled tube‐in‐tube structure. Ru and Mn are homogeneously distributed on the RM‐TIT without distinctive phase separation. In OER performance, the onset potential of RM‐FIT and RM‐TIT are lower than those of commercial Pt/C electrodes. In ORR performance, the half‐wave potential (*E*_1/2_) of RM‐TIT is −0.08 V (vs Hg/HgO electrode), which is close to that of the outstanding ORR catalyst Pt/C (−0.06 V vs Hg/HgO electrode). When RM‐FIT and RM‐TIT were used in a real Li−O_2_ battery system, the air electrodes exhibited increased overpotential characteristics and stable cyclability over 100 cycles. This one‐dimensional nanostructure catalyst is conducive to electron transport and substance diffusion, and the presence of RuO_2_ effectively regulates the electronic structure of Mn_2_O_3_, resulting in excellent bifunctional catalytic activity.

The interfacial effect can remarkably facilitate electron transfer and expose active sites. Thus designing the heterostructure is a promising approach to enhancing the catalytic performance of catalysts.^[^ ^34^ ^]^ Through the electrospinning strategy, the obtained porous NiO/NiCo_2_O_4_ NFs show excellent electrocatalytic performance due to the heterostructure with abundant interface‐related active sites and electronic transmission channels.^[^ ^17c^ ^]^ The interfacial engineering resulted in rich Ni^3+^ and Co^3+^ species in NiO/NiCo_2_O_4_ nanofiber and the coupling interface formed by Ni^3+^ and Co^3+^ enhances the intrinsic activity of the catalyst. The results of density functional calculation (DFT) demonstrate that the formation of chemical bonds between NiO and NiCo_2_O_4_ could accelerate the charge transfer, therefore, the porous NiO/NiCo_2_O_4_ NFs display extremely efficient and durable performances of OER and ORR in KOH solution. The NiO/NiCo_2_O_4_‐based Zn−air battery possesses outstanding specific capacities of 814.4 mA h g^−1^, and remarkable cycling stability of 175 h, and the flexible Zn−air battery shows good flexibility and long cycling life of 14 h.

In addition to proton exchange membrane fuel cells (PEMFC) and metal‐air batteries, as alternative power sources, direct hydrazine fuel cells (DHFCs) as well as direct alcohol fuel cells (DAFCs), have also received attention in recent years.^[^ ^35^ ^]^ Growing research focuses on the fabrication of transition metal oxide CNFs catalysts via electrospinning technology. The electrospun CNFs catalysts with transition metal oxides (e.g. NiO, CuO, and Co_3_O_4_) were also used to prepare CNFs catalysts for electro‐catalytic oxidation of ethylene glycol,^[^ ^36^ ^]^ methanol,^[^ ^17d^ ^]^ and hydrazine.^[^ ^37^ ^]^ This fibrous nanoreactor with 1D structure brings in higher surface area and greater numbers of active sites and obviously improves the catalytic activity and stability of CNFs catalysts.

#### 3.1.2 Multi‐element transition metal oxides

Owing to the higher catalytic activity, flexible structure, and low cost, perovskite oxides have great potential in various energy‐related applications as an efficient electrocatalyst. However, only perovskite catalysts with large particle sizes, small surface area, and few morphological features could be obtained through traditional synthetic methods, and this drawback results in limited catalytic activity.^[^ ^38^ ^]^ In this sense, the electrospinning strategy can provide a reliable solution for preparing perovskites with tunable morphologies and complex compositions.^[^ ^39^ ^]^ In addition, the electrospun one‐dimensional perovskite nanostructures exhibit higher catalytic activity and long‐term stability in an alkaline solution which offers a cost‐effective alternative to noble metal‐based electrocatalysts.^[^ ^40^ ^]^

**FIGURE 3:**
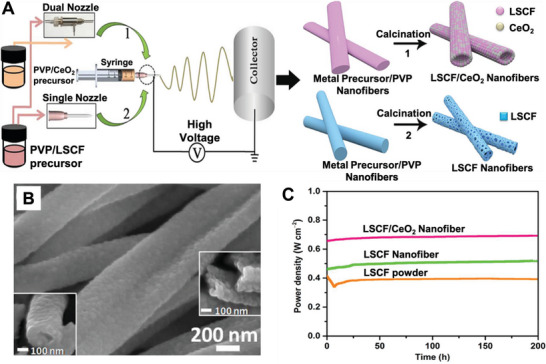
Preparation and electrocatalytic performance of LSCF/CeO_2_ and LSCF NFs. A) The preparation process of the LSCF/CeO_2_ and LSCF nanofibers by electrospinning. B) SEM image of LSCF/CeO_2_ NFs sintered at 800°C for 2 h. C) Stability test of the single cells at 600°C (under a constant voltage of 0.4 V). Reproduced with permission.^[^ ^41^ ^]^ Copyright 2019, American Chemical Society.

Through the coaxial electrospinning technique, Zhang et al. synthesized heterostructured fibrous cathode materials consisting of La_0.6_Sr_0.4_Co_0.2_Fe_0.8_O_3−_ *_δ_* (LSCF) and CeO_2_ NPs (Figure 3).^[^ ^41^ ^]^ Different from the core–shell structure, the random arrangement of LSCF and CeO_2_ nanoparticles formed unique heterostructured nanofibers. The heterostructure creates abundant hetero‐interfaces between LSCF and CeO_2_ and provides a continuous route for efficient mass/charge transport. Compared with the single LSCF powder, the ORR activity and durability of LSCF/CeO_2_ composite nanofibers in solid oxide fuel cells (SOCF) are significantly enhanced. This improvement of ORR activity is due to the continuous route provided by the electrospun fibers for efficient mass/charge transport and the interdiffusion of La and Ce at the hetero‐interface resulting in the formation of more oxygen vacancy. The fuel cells based on LSCF/CeO_2_ composite electrodes maintained outstanding long‐term stability (0.4 V for ∼200 h at 600°C) which demonstrates the microstructure design of these heterostructured nanofibers for LSCF/CeO_2_ could be extremely effective for improving ORR performance.

**FIGURE 4:**
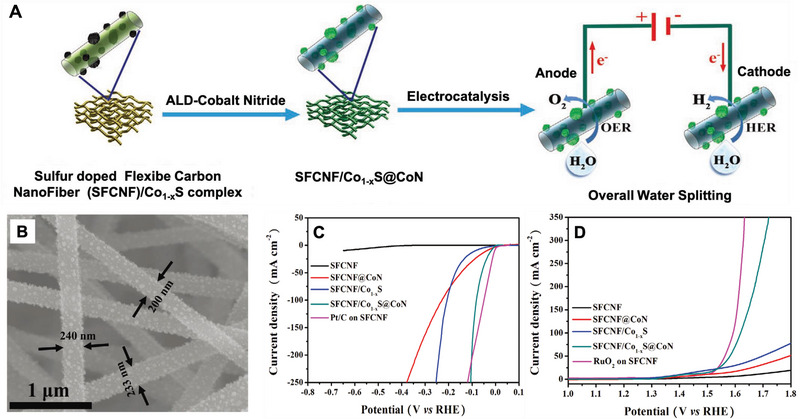
Preparation and electrocatalytic performance of SFCNF/Co_1−_ *_x_* S@CoN. A) Preparation of SFCNF/Co_1−_ *_x_* S@CoN composite. B) SEM image of SFCNF/Co_1−_ *_x_* S @CoN composite. C) HER performance: LSV curves with a scan rate of 10 mV s^−1^, and 91% *iR* correction. D) OER performance: LSV curves with a scan rate of 10 mV s^−1^ and 91% *iR* correction. Reproduced with permission.^[^ ^42b^ ^]^ Copyright 2020, Wiley‐VCH.

The 1D transition metal catalysts prepared by the electrospinning process have an ideal specific surface area, porosity, and conductivity, and exhibit excellent OER, ORR, and even HER catalytic performance in an alkaline environment. However, in the development of water‐splitting technologies, fabricating the catalysts with excellent OER and HER activity in acidic electrolytes is the crucial bottleneck.^[^ ^42^ ^]^ Therefore, the development of economic and abundant transition‐metal‐based HER and OER catalysts, especially the bifunctional electrocatalysts in acidic solutions are urgently requested.^[^ ^42a,b^ ^]^ Guo et al. synthesized a SFCNF/Co_1−_ *_x_* S@CoN catalyst for overall water‐splitting.^[^ ^42b^ ^]^ The S‐doped flexible carbon nanofiber (SFCNF) matrix, Co_1−_ *_x_* S nanoparticles, and CoN coatings construct the composite nanofiber through the combination of electrospinning and atomic layer deposition (ALD) strategy (Figure 4). Component of Co_1−_ *_x_* S nanoparticles itself is active site for HER and OER reactions. The CoN thin film grown on the Co_1−_ *_x_* S defects through the ALD strategy could induce a synergistic effect between the metal sulfide and the metal nitride. This synergistic effect can effectively promote the electron transfer between the current collector and the catalyst. Additionally, the superior electrical conductivity of the SFCNF substrate can improve the diffusion of the electrolyte by enhancing the contact between the reactants and active sites. Therefore, this composite material exhibits strong HER and OER activities both in alkaline and acidic media. Specifically, the combination of electrospun fibers and ALD technology (or other techniques for preparing nanomaterials) can also be further studied to prepare novel nanomaterials with advanced structures. The strategy will provide a promising alternative for overall water‐splitting and other renewable energy applications.

### 3.2 Metalcarbon nanofiber composite catalysts

**FIGURE 5:**
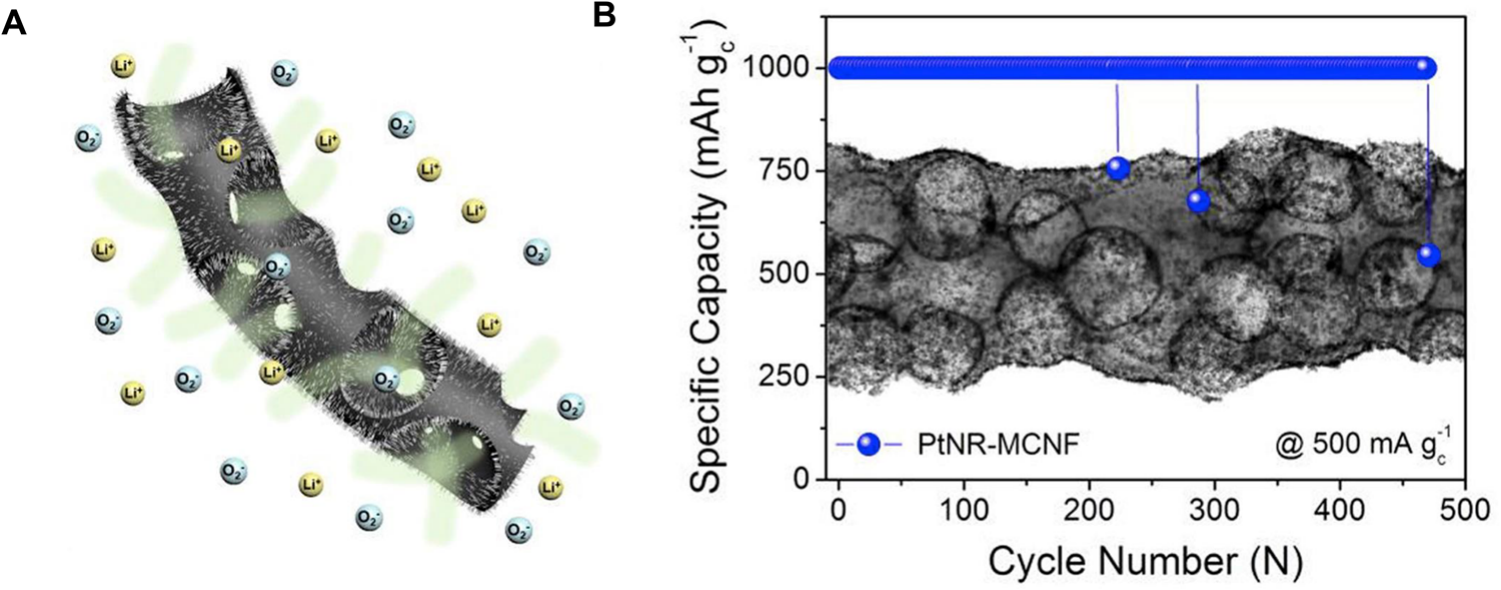
Free‐standing cathodes with Pt nanorods decorated carbon nanofiber for Li‐O_2_ battery. A) Schematic illustrations of PtNR‐MCNF. B) Plot of specific capacity as a function of cycle number and TEM image of PtNR‐MCNF. Reproduced with permission.^[^ ^43a^ ^]^ Copyright 2019, Elsevier.

Due to the advantages of uniform distribution, controllable morphology, and good conductivity of free‐standing electrospun carbon nanofibers, the combination of noble‐metal (such as Pt or Ru NPs) with carbon nanofibers has great potential in improving stability and broadening application fields (Figure 5). Noble metal‐based carbon nanofibers are widely used as electrocatalysts due to the exceptional activity.^[^ ^43^ ^]^ However, the high price, limited durability, scarce crustal abundance, low bifunctional activity, as well as poor resistance to poisoning restrict their further large‐scale commercial applications.

To solve the above problem, recent research found that the introduction of transition metal into carbon nanofiber could also significantly improve catalytic performance, providing a possibility for exploiting novel cost‐effective electrocatalysts with high efficiency. And the metal species (nanoparticles and/or metal atoms) coordinated with heteroatom atoms (mainly N) could provide modified electronic structure and new active sites.^[^ ^31^, ^44^ ^]^ Current strategies mainly including two methods: (1) adding metal salts (such as acetate, acetylacetonate, etc.) directly into the electrospun solution,^[^ ^45^ ^]^ (2) incorporating pre‐designed functional “nanofillers” (such as metal phthalocyanine, metal‐organic framework, etc.) into the polymer solution to prepare metal‐containing composite carbon nanofibers.^[^ ^27^, ^46^ ^]^ As the polymer (such as PAN) used for electrospinning is full of functional groups, which could have special coordinate effect with the metal atom. And during the follow‐up carbonization process after electrospinning, the metal species tend to be agglomerated into large metal nanoparticles at high temperature (> 700°C), while the composite fibers could prevent the metal from agglomeration trend and inherit good conductivity of the carbon nanofiber.

Yang and co‐workers prepared the N‐doped carbon nanofibers with NiCo alloy NPs decorated as a bifunctional catalyst through pyrolysis of PVP‐PAN/metal nitrate hexahydrate fibers.^[^ ^45c^ ^]^ It was found that the content of NiCo alloy NPs and the form of the prepared NiCo@N‐C could be changed by adjusting the amount of metal salts, and the moderate doping of NiCo alloy NPs loading gives the excellent ORR/OER electrocatalytic performances. Accordingly, the NiCo@N‐C‐2 exhibited a comparable ORR and OER (*E*_1/2_ = 0.81 V vs. RHE and *E*_j = 10_ = 1.76 V vs. RHE, respectively) to the Pt/C and RuO_2_ catalyst (0.80 and 1.76 vs. RHE, respectively). Meanwhile, the NiCo@N‐C‐2 exhibits the smallest Tafel plot slopes for both ORR and OER (−65 and 98 mV dec^−1^, respectively). The excellent performance of the prepared NiCo@N‐C‐2 catalyst is due to the synergetic effects of the N‐doped carbon nanofiber and metal NPs, which improves the conductivity, increases C═C, graphitic‐N/pyridinic‐N ratio, and forms the highly active metal hydroxylation.

**FIGURE 6:**
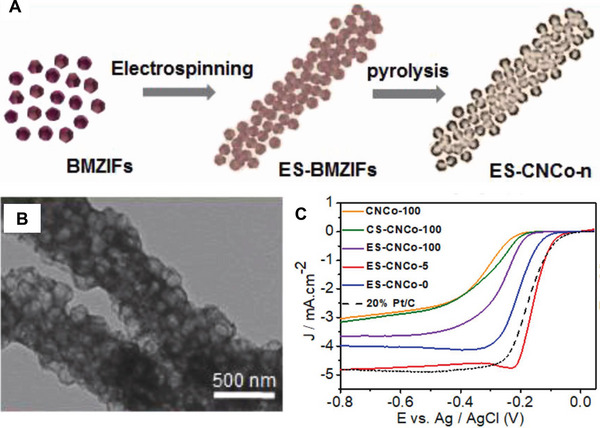
Preparation and electrocatalytic performance of ES‐CNCo‐n. A) Synthetic process illustration diagram and B) TEM image of ES‐CNCo‐5. C) LSV curves of prepared fibrous materials and Pt/C (0.1 M KOH). Reproduced with permission.^[^ ^47^ ^]^ Copyright 2019, Elsevier.

Yu et al. fabricated the porous carbon nanofibers with both Co and N co‐doped via the thermal treatment of MOF nanofibers. The MOF NFs were prepared through electrospinning of bimetallic zeolitic imidazolate framework nanoparticles (BMZIFs) (Figure 6).^[^ ^47^ ^]^ The obtained BMZIFs‐containing nanofibers with a thin PAN layer wrapped on the nanoparticles, the structure of which could prevent the aggregation and fusion of Co during the follow‐up pyrolysis. And the derived fibrous porous carbon materials ES‐CNCo‐n possessed hierarchical pores and regular shape, embedded with highly‐dispersed Co nanoparticles wrapped by graphitic carbon shell. Through comparison, it was found that samples ES‐CNCo‐5 (Zn/Co molar ratio is 5) displayed excellent ORR performances comparable to the Pt/C catalyst. In an alkaline electrolyte (0.1 m KOH), the catalyst showed onset and *E*_1/2_ potentials of −0.08 and −0.155 V vs. Ag/AgCl, respectively, which are close to the Pt/C catalyst (−0.07 and −0.168 V vs. Ag/AgCl). And in 0.5 m H_2_SO_4_ solution, the catalyst also exhibited excellent performance with the onset and *E*_1/2_ potentials of 0.52 and 0.43 V vs. Ag/AgCl, respectively. Additionally, the obtained catalyst exhibited good stability and methanol tolerance. Compared with the non‐electrospun sample, the high ORR performance of the catalyst was attributed to the high surface area with uniform Co, N dopants, and the 1D porous structure promotes mass transfer with more exposed active sites.

At present, non‐precious metal atom and nitrogen co‐doped carbon materials (M‐N‐C) have become a major alternative to precious metal catalysts for electrocatalytic activity promotion due to their maximum atomic utilization, strong metal‐substrate interaction, unique size quantum effects, and extraordinary catalytic performance.^[^ ^48^ ^]^ The research showed that the performance of M‐N‐C is closely related to their dispersion state, configuration, and interactions with the support. Therefore, it is necessary to construct a synthesis approach with atomic‐level precision. Besides, although great successes have been achieved in the development of active M‐N‐C, most of the currently used catalysts still suffer from their powdery form, and the use of extra organic binder and conductive current collector for electrode fabrication may not only cause the blockage of active catalytic sites and deteriorates the electrical conductivity, but also affect the mass energy density and the cycling life of the device. In addition, attempts have been made to embed M‐N‐C in CNFs for catalytic film integration, which could help with the mass energy density and the cycling life of the energy device.^[^ ^44^, ^46^, ^49^ ^]^

**FIGURE 7:**
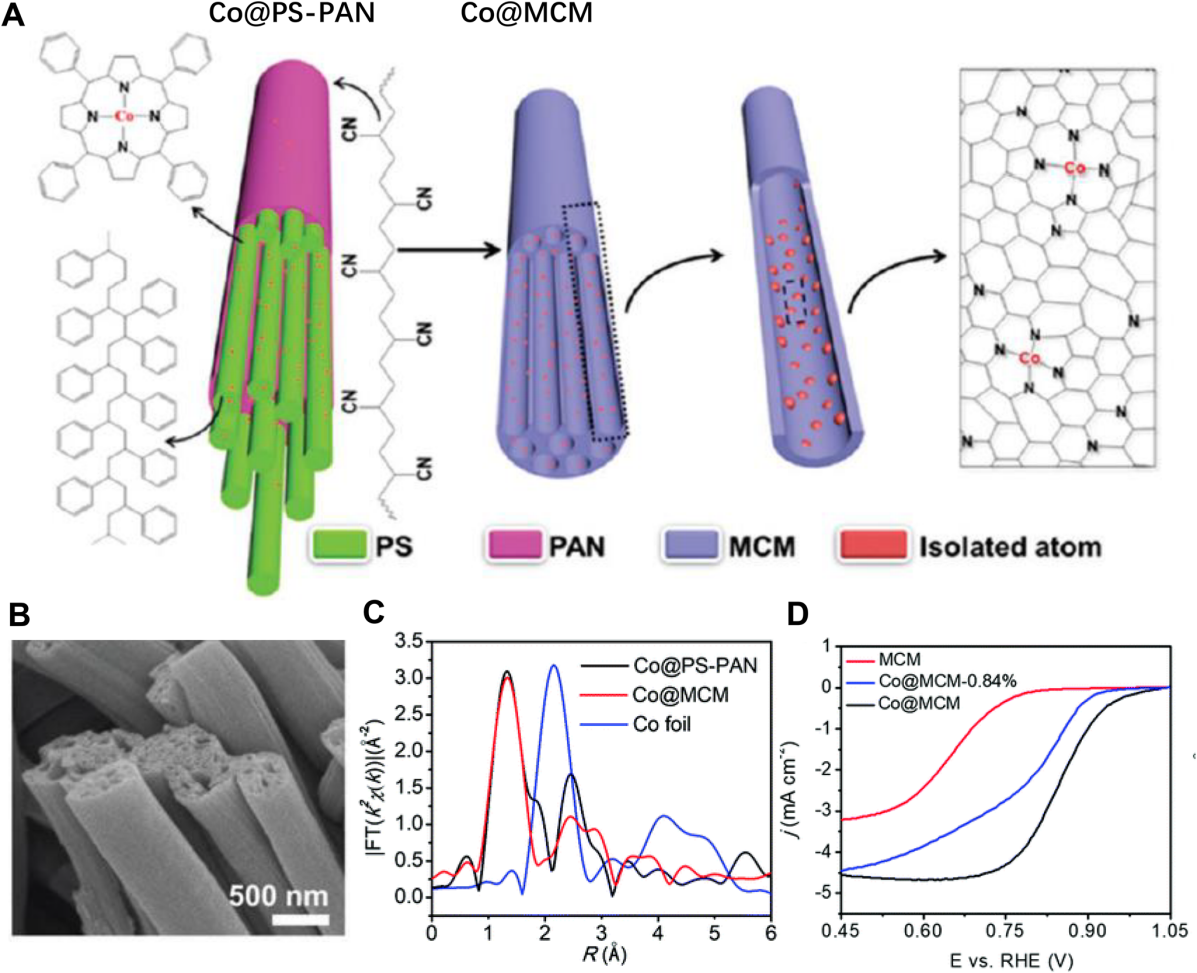
Preparation and electrocatalytic performance of Co@MCM. A) Synthetic process illustration diagram and B) FESEM image of Co@MCM, C) Co K‐edge EXAFS spectra, D) ORR LSV curves of samples (0.1 m KOH). Reproduced with permission.^[^ ^27a^ ^]^ Copyright 2018, Royal Society of Chemistry.

Lou and his co‐workers reported a modular method for preparing interconnected multichannel carbon matrix with atomically dispersed Co atoms (Co@MCM, Co content of about 1.4 wt%) based on a pre‐designed configuration (Figure 7).^[^ ^27a^ ^]^ The Co EXAFS spectra of obtained Co@MCM were similar to the Co@PS‐PAN precursor, demonstrating that the pre‐designed configuration of Co‐center is well maintained after pyrolysis. Besides, further characterization results showed that the homogeneously decorated CoN_4_ units together with the 3D porous carbon matrix endowed the catalyst with good activity. In 0.1 m KOH, LSV curves displayed that the Co@MCM exhibits better ORR performance compared to the MCM both in terms of *E*_onset_ (0.95 and 0.78 V vs. RHE) and *E*_1/2_ (0.86 and 0.67 V vs. RHE). By reducing the loading amount of Co to 0.84%, both *E*_onset_ and *E*_1/2_ the obtained Co@MCM‐0.84% exhibited a negative shift, indicating that the ORR activity was closely related to the number of Co active sites.

Wu and co‐workers developed a porous carbon nanofiber‐based Co catalyst (Co‐N‐PCNF) by electrospinning Co‐doped ZIFs into specific PAN‐PVP polymers.^[^ ^44d^ ^]^ Compared to conventional Co‐N‐C catalyst, the hierarchical and porous fibrous structures of Co‐N‐PCNF possessed more exposed active sites and better electron conductivity, which played a significant role in increasing the ORR activity (*E*_onset_ = 0.95 V vs. RHE and *E*_1/2_ = 0.81 vs. RHE in 0.5 m H_2_SO_4_). The results of macroscopic properties by using nano‐CT imaging showed that 3D structure and ionomer distribution mainly affected the PGM‐free cathodes in membrane electrode assemblies of PEMFCs. Besides, the stability and durability of the catalyst were also enhanced due to the high graphitization of the carbon matrix, which is beneficial to the corrosion resistance. In a practical H_2_/air battery assembled with the as‐prepared catalyst, a power density as high as 0.4 W cm^−2^ was achieved with good stability.

### 3.3 Preparation of one‐dimensional **Iridium‐based** catalyst

Hydrogen energy is recognized as clean energy source and recent studies mainly focus on the hydrogen procedure process in large quantities and at a relatively low cost.^[^ ^50^ ^]^ Electrochemical reduction of water is generally chosen as one of the main approaches for the production of molecular hydrogen.^[^ ^51^ ^]^ In electrolytic water splitting, the HER is the cathode half‐reaction, and in acidic media, the HER can be described as:^[^ ^50^, ^51^, ^52^ ^]^

(1) $$\begin{array}{cc} 4H^{+}+4e^{-}\to 2H_{2} & E^{\circ }=0\;V\left(\mathrm{vs}\;\text{SHE}\right) \end{array}$$

For proton reduction process, a suitable catalyst is a key point for minimizing the overpotential and maximizing the efficiency of the reaction.^[^ ^51^ ^]^ Noble‐metal catalysts seem to be a desirable choice, however, the high cost limited their application in industrial processes.^[^^]^ Recent investigations reported that metal alloys,^[^ ^54^ ^]^ metal oxides,^[^ ^55^ ^]^ metal dichalcogenides,^[^ ^56^ ^]^ and even enzymes^[^ ^57^ ^]^ could be used as electrocatalysts. In particular, iridium (Ir) possesses corrosion resistivity in acidic conditions^[^ ^51^ ^]^ and the adsorption energy of Ir‐H_ads_ is close to that of Pt‐H_ads_,^[^ ^58^ ^]^ thus the Ir‐based electrocatalysts attracted more attention.^[^ ^58^, ^59^ ^]^

**FIGURE 8:**
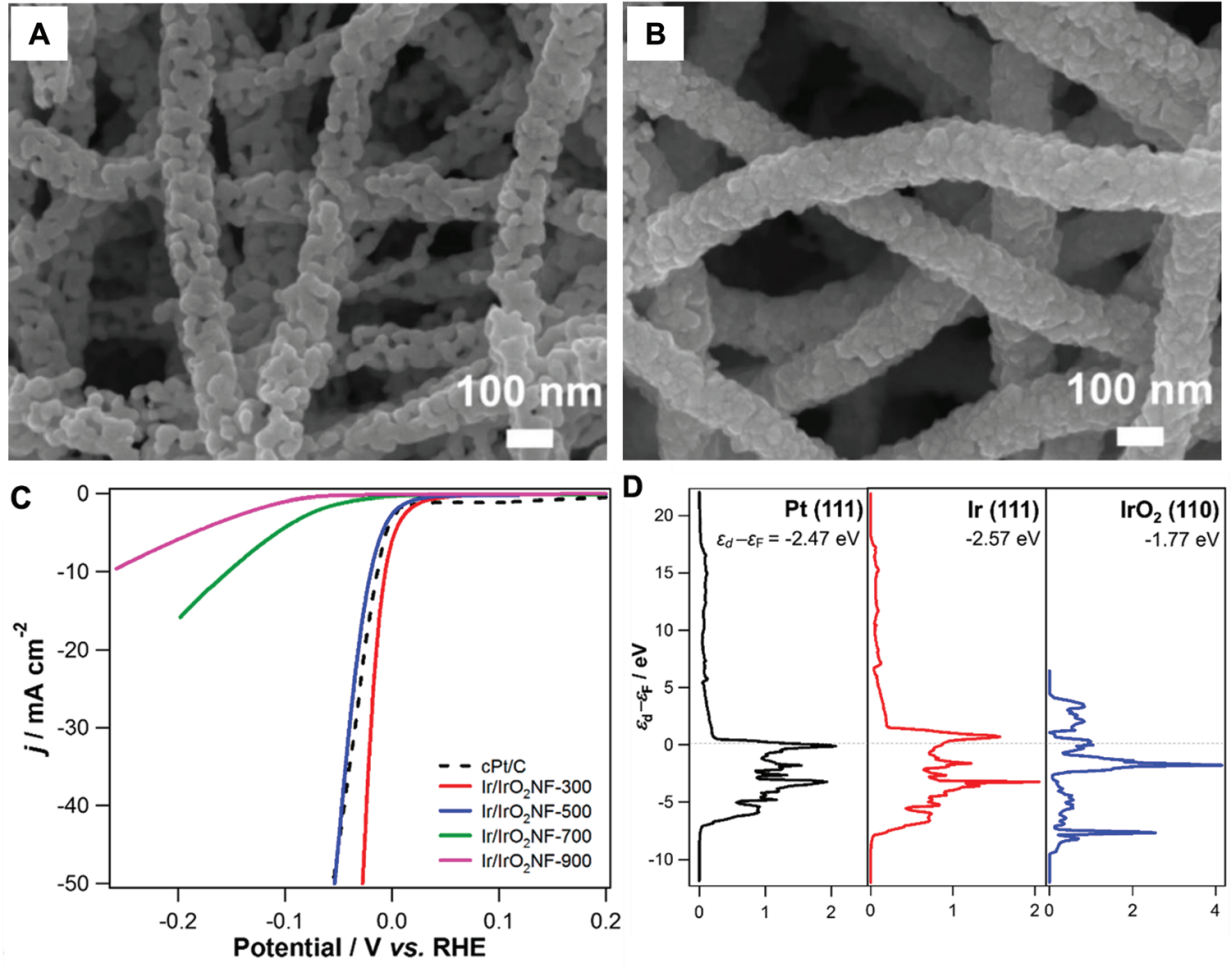
Morphology and electrocatalytic performance of Ir/IrO_2_NFs. FE‐SEM images of Ir/IrO_2_NFs under thermal treatment at A) 300°C and B) 900°C for 2 h in air. C) RDE polarization curves in a cathodic potential window of the different Ir/IrO2 NFs and cPt/C in 1.0 m H_2_SO_4_. (The potential sweep rate is 5 mV s−1; the electrode rotation rate is 900 rpm). D) Electronic density of states projected onto the d‐band for different metal reciprocal cells (*ε*d − *ε*F; relative to the Fermi level). Reproduced with permission.^[^ ^51b^ ^]^ Copyright 2019, American Chemical Society.

1D nanofiber catalysts produced by the electrospinning process are also a promising approach for fabricating Ir‐based catalysts.^[^ ^60^ ^]^ Compared to wet synthesis, electrodeposition, and chemical vapor deposition processes, the electrospinning technique is a powerful tool to construct Ir‐based electrocatalysts with diverse morphologies and compositions.^[^ ^61^ ^]^ Kim et al.^[^ ^51b^ ^]^ synthesized the electrospun iridium/iridium oxide nanofibers (Ir/IrO_2_NFs) (Figure 8). At low calcination temperatures (300°C and 500°C), the networks of individual distinctive nano pebbles formed porous lumpy structured Ir/IrO_2_NFs (Figure 8A), while the higher annealing temperature resulted in Ir/IrO_2_NFs with densely packed morphology which is composed of less distinguishable pebble building blocks (Figure 8B). In terms of catalytic performance, the HER activity of Ir/IrO_2_ NFs in 1 mol L^−1^ H_2_SO_4_ solution on the overpotential basis is calculated to be in the following order: Ir/IrO_2_NF‐300 > commercial Pt/C > Ir/IrO_2_NF‐500 > Ir/IrO_2_NF‐700 > Ir/IrO_2_NF‐900. The Ir/IrO_2_NF‐300 catalyst showed outstanding HER activity with the largest cathodic current, the least onset potential (∼0.0 V), the highest TOF, and smaller Tafel slope (30 mV dec^−1^). On the other hand, the DFT calculation exhibited that the value of Fermi level (*ε*_d_ − *ε*_F_), *E*_ads_ (adsorption energy) of H atom, and *d*_M−H_ (distance between catalyst M surface and an H atom adsorbate) of Ir(111), rather than IrO_2_(110), are very close to those of Pt(111). Therefore, these results illustrate that Ir rather than IrO_2_ is an excellent HER catalytic platform which points out a clear direction for the preparation of high‐activity HER catalysts.

**FIGURE 9:**
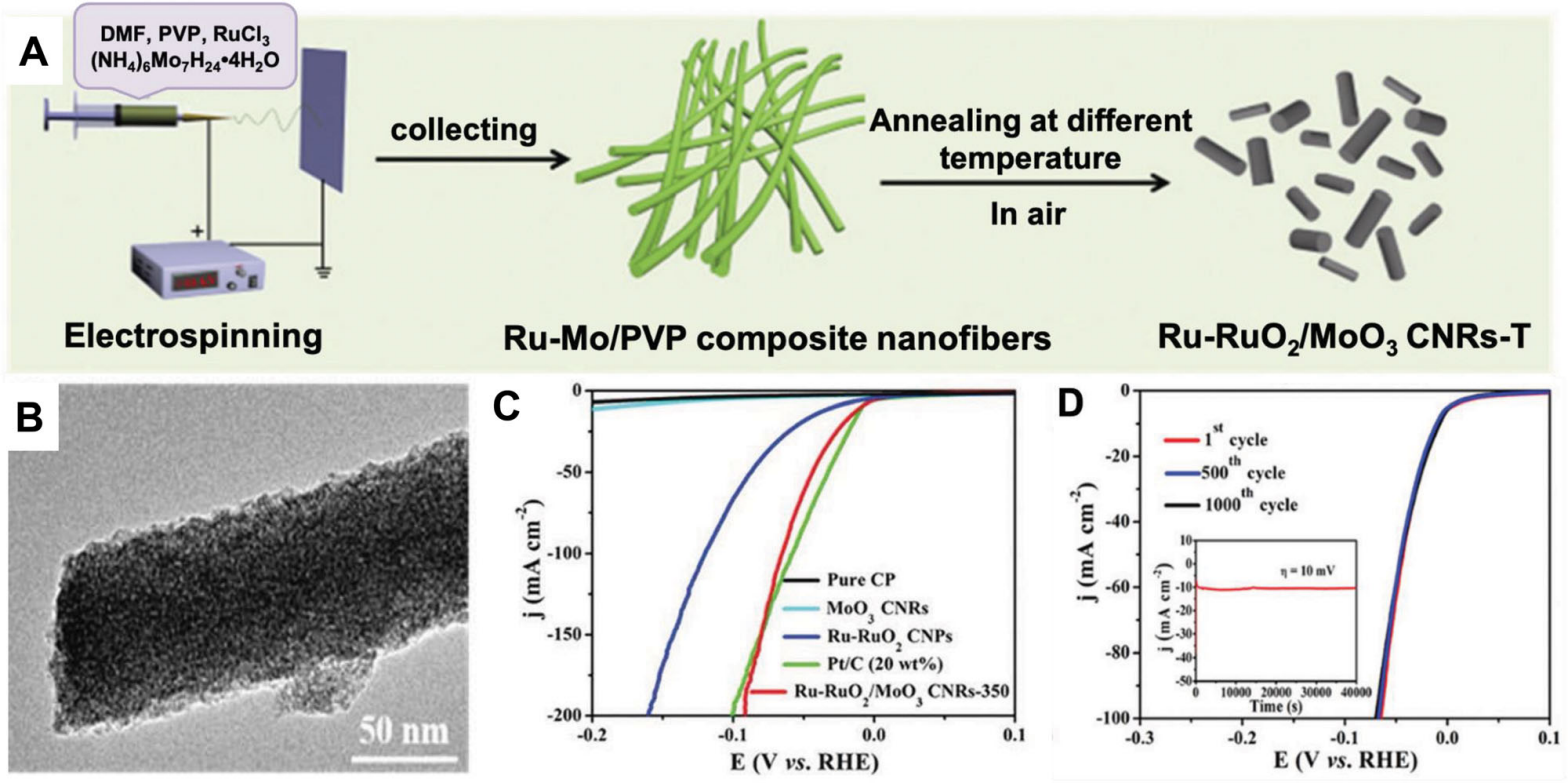
Preparation and electrocatalytic performance of Ru–RuO_2_/MoO_3_CNRs‐T. A) The fabricating process of Ru–RuO_2_/MoO_3_CNRs‐T. B) TEM image of Ru–RuO_2_/MoO_3_ CNRs‐350. C) Polarization curves at 0.15 V versus RHE as a function of the scan rate for MoO_3_ CNRs, Ru–RuO_2_CNPs, and Ru–RuO_2_/MoO_3_ CNRs‐350. D) Polarization curves of Ru–RuO_2_/MoO_3_ CNRs‐350 initially, after 500 and 1000 CV cycles (inset: chronoamperometry curves of the Ru–RuO_2_/MoO_3_ CNRs‐350 catalyst at *Z* = 10 mV). Reproduced with permission.^[^ ^66^ ^]^ Copyright 2020, Royal Society of Chemistry.

IrO_2_^[^ ^58^, ^59^ ^]^ and RuO_2_^[^ ^58^, ^59^, ^62^ ^]^ are promising candidates as efficient HER catalysts owing to the stability of IrO_2_^[^ ^58^ ^]^ and the resistance of ruthenium oxide (RuO_2_) under potential deposition poisoning in the presence of metal ions.^[^ ^59^, ^63^ ^]^ Cho et al.^[^ ^64^ ^]^ fabricated a series of Ir*_x_* Ru_1−_ *_x_* O*_y_* (*y* = 0 or 2) NFs, and Ir_0.80_Ru_0.20_O*_y_* exhibited superior HER activity even better than commercial Pt (cPt) and Ir (cIr) in alkaline solution. The potentials of cPt and cIr shifted toward a more negative direction after 10,000 s. On the contrary, the potential of Ir_0.80_Ru_0.20_O*_y_* shifts toward a more positive direction during a constant current supply, demonstrating their cathodic activation of HER activities. Electrochemical stability is an essential parameter for evaluating the HER performance of catalysts from an economic point of view. Although the noble metal ruthenium has better catalytic activity for water electrolysis than iridium, the RuO_2_ can be overoxidized into dissolvable RuO_4_ at applied OER potentials. Additionally, RuO_2_ suffer from corrosion in acidic condition which prevents its wide application in acidic media.^[^ ^65^ ^]^ Recently, Fan et al.^[^ ^66^ ^]^ prepared a series of Ru‐RuO_2_/MoO_3_ embedded carbon nanorods (Ru‐RuO_2_/MoO_3_ CNRs) catalyst under various annealing temperatures (Figure 9). The Ru–RuO_2/_MoO_3_ CNRs‐350 (the calcination temperature is 350) catalyst possesses excellent HER activity due to the synergetic effect between Ru–RuO_2_ and MoO_3_. Particularly, the HER performance of the Ru–RuO_2/_MoO_3_ CNRs‐350 catalyst is comparable to that of cPt/C catalysts, and even superior to it at higher current densities. The stability test shows Ru–RuO_2/_MoO_3_ CNRs‐350 catalyst maintained their original morphology and no obvious change in the current density was observed at the constant overpotential of 10 mV for more than 40,000 s. Meanwhile, the current density of Ru‐RuO_2_/MoO_3_ CNRs‐350 catalyst exhibits an insignificant decay after 500 and 1000 cyclic voltammetry tests. The superior capability of Ru‐RuO_2_/MoO_3_ CNRs‐350 catalyst benefits from the electronic conductivity and larger electrochemical surface area of the electrospun fibers as well as the synergetic effect between Ru and Mo components. For this reason, this approach of fabricating HER catalysts could also be inspired to prepare iridium‐based catalysts with excellent stability.

**FIGURE 10:**
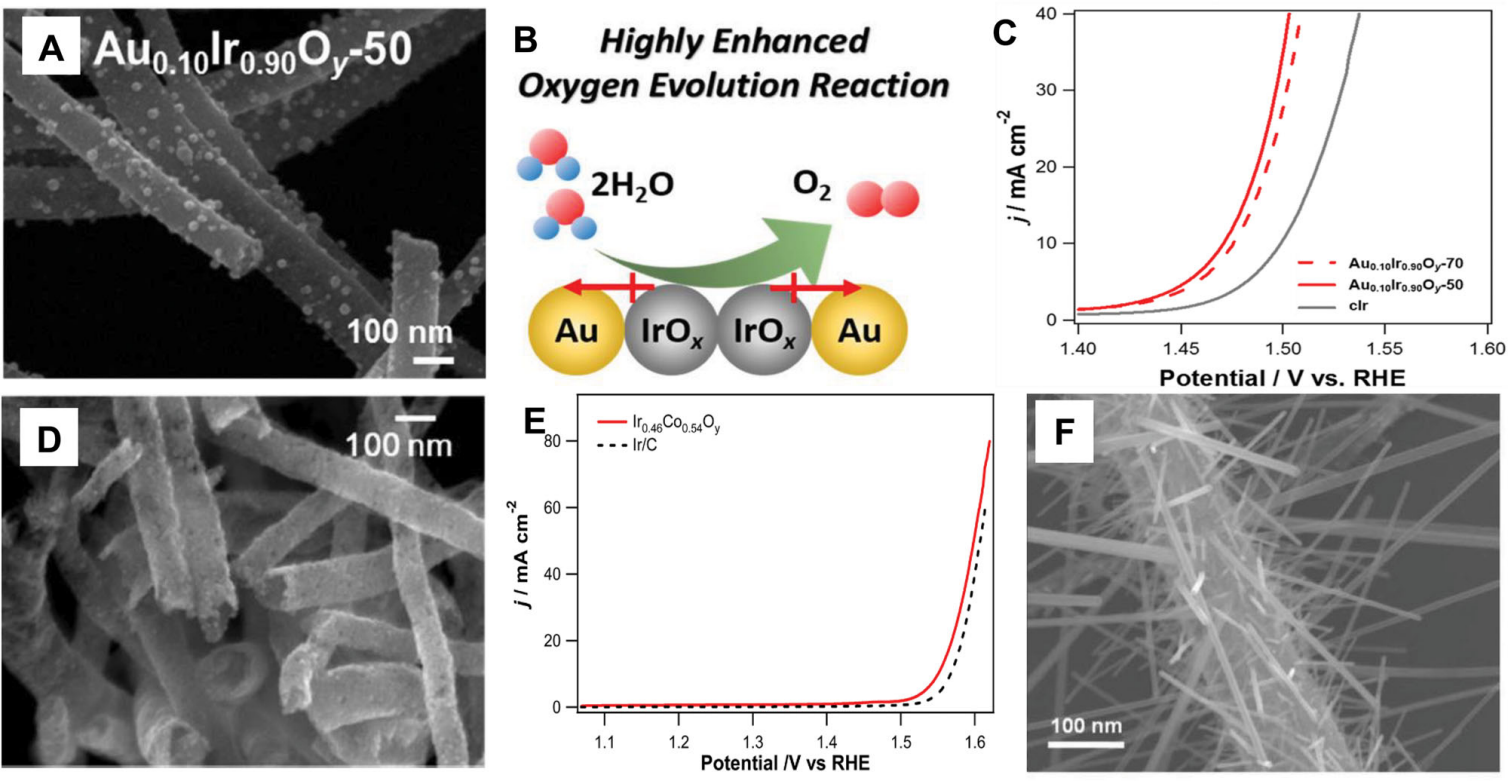
Morphology and electrocatalytic performance of Ir‐based NFs. A) SEM image of Au_0.10_Ir_0.90_O*_y_*‐50. B) Schematic diagram of electrophilic OER active sites. C) RDE curves of Au_0.10_Ir_0.90_O*_y_*‐70, Au_0.10_Ir_0.90_O*_y_*‐50, and Ir/C for OER obtained in Ar‐saturated 0.5 m H_2_SO_4_ (the rotation speed is 1600 rpm; the scan rate is 10 mV s^−1^). D) SEM image of Ir_0.46_Co_0.54_O*_y_*. E) Comparison between Ir_0.46_Co_0.54_O*_y_* and Ir/C for the OER in Ar‐saturated 1 m NaOH aqueous solution (the rotation rate is 1600 rpm; the scan rate is 10 mV s^−1^). F) SEM image of hierarchically grown RuO_2_ nanowires on electrospun IrO_2_ nanofibres via a simple vapor phase transport process at 650°C for 2 h under He–O_2_ (450 sccm/15 sccm) mixed carrier gas flow. A–C) Reproduced with permission.^[^ ^61a^ ^]^ Copyright 2019, American Chemical Society. D,E) Reproduced with permission.^[^ ^61b^ ^]^ Copyright 2017, American Chemical Society. F) Reproduced with permission.^[^ ^68^ ^]^ Copyright 2013, Royal Society of Chemistry.

Ir‐based catalysts are also used in the OER. Moon et al.^[^ ^61a^ ^]^ synthesized hierarchical nanostructured Au*_x_* Ir_1‐_ *_x_* O*_y_* NFs (*x* = 0.05, 0.10, or 0.33) with various compositions of gold (Au) and iridium (Figure 10A–C) via simple single‐step electrospinning. Among them, Au_0.10_Ir_0.90_O*_y_*‐50 NFs (50 is the volume % of ethanol in the ethanol/DMF mixed solvent) with 10% Au loading could produce higher OER activity and stability compared to Ir/C catalysts. Yu et al.^[^ ^61b^ ^]^ fabricated one‐dimensional tubular Ir*_x_* Co_1−_ *_x_* O*_y_* NFs while Ir_0.46_Co_0.54_O*_y_* NF possesses excellent OER activity even superior to cIr/C catalysts (Figure 10D,E). In the alkaline condition, Ir_0.46_Co_0.54_O*_y_* NF maintained high stability during 1000 iterative OER scans. Importantly, the mixture of Ir and Co at an appropriate ratio exhibits much higher OER catalytic performance than pure Ir oxide owing to the synergetic effect between Ir and Co oxides.^[^ ^67^ ^]^ This combination of Ir and Co oxides reduces the loading of noble metal Ir, which reduces the catalyst's cost effectively.

However, these approaches of one‐dimensional Ir‐based catalysts fabrication are limited to the experimental research level. The electrospinning efficiency still needs to improve and the equipment cost should be reduced, so as to really improve the economic benefits of electrospinning to reach the standard of mass production in the future. Some approaches such as coaxial electrospinning are also needed for further development for fabricating more advanced fibrous structures, such as core–shell, multilayer, and multi‐component nanofibers. At the same time, the electrospinning process could be combined with other technologies (such as heat treatment, plasma treatment, chemical grafting, and solution deposition) to further improve the diversity of the fiber structure. By combining the electrospinning and vapor phase transport process, Lee et al.^[^ ^68^ ^]^ first reported a facile synthesis route of hierarchically grown single crystalline metallic RuO_2_ nanowires on the electrospun IrO_2_ nanofibers (Figure 10F). The IrO_2_ NF consists of the continuous connection of the particle‐like IrO_2_ with a diameter of 5–10 nm. The presence of diverse crystalline planes of the IrO_2_ crystal structure demonstrates the polycrystalline nature of a nanofiber, and its catalytically active surface is fully exposed. A single RuO_2_ nanowire grows along the (011) direction on the IrO_2_ nanofiber, which possesses a single crystalline nature with no sign of any defects, dislocations, and amorphous over‐layers. The synthetic strategy of this one‐dimensional heterostructure promotes the uniform combination of two metal oxides, which will be beneficial to the improvement of the catalytic performance of HER and OER. The reasonable transformation of existing equipment and further design of various electrospinning jet devices and collectors will effectively control the orientation of nanofibers. In addition, the development of more advanced electrospinning technology and combination with other methods will better control the structure and morphology of nanofibers, resulting in ordered and diverse nanofiber catalysts to further improve the activity and stability of fibrous catalysts.

### 3.4 Preparation of antimony tin oxide (ATO) nanofibers through electrospinning process

The chemical composition, surface area, and physical properties of materials can affect the activity of the catalyst.^[^ ^69^ ^]^ Disperse fewer amounts of expensive catalysts on suitable support with a large surface area could construct the supported catalyst and this strategy could effectively enhance the mechanical properties of the catalyst. The desirable properties of excellent catalyst support are outstanding mechanical properties, inertness, stability, porosity, and high surface area.^[^ ^69^ ^]^ In this regard, various conductive supports like Ti*_n_* O_2_ *_n_*_‐1_ (4 < *n* < 9),^[^ ^70^ ^]^ NbC,^[^ ^71^ ^]^ TiC,^[^ ^72^ ^]^ and ATO^[^ ^73^ ^]^ were chosen as electrocatalyst supports. Remarkably, ATO is a promising support material owing to its superior electrical conductivity and stability.^[^ ^74^ ^]^ However, the low surface area of ATO materials largely restricted the performance of ATO‐supported catalysts. The 1D ATO‐modified carbon nanofibers obtained by electrospinning can effectively surmount this shortcoming and improve the charge transport properties in the catalyst layer.^[^ ^75^ ^]^ For example, the electrospun ATO nanofibers could be used as the porous electrodes^[^ ^76^ ^]^ and the negative electrode of Li‐ion battery^[^ ^77^ ^]^ since the ATO nanofibrous structure can extremely enhance the electrical conductivity of the composite catalysts. In addition, through the electrospinning process, Xu et al.^[^ ^78^ ^]^ developed novel catalyst support, Sb‐SnO_2_ nanowire. The pore structure of the Sb‐SnO_2_ nanowire provides a more accessible surface area for the electrolyte. After IrO_2_ catalysts are loaded onto the Sb‐SnO_2_ nanowire, the OER catalytic activity of the Sb‐SnO_2_ nanowire‐supported IrO_2_ catalyst exhibits about three times higher than that of the pure IrO_2_ catalyst. The electrospun ATO nanofibers with excellent properties were also used as the support of Pt‐based catalyst for improving the electrocatalysis performance of the catalyst.^[^ ^75^, ^79^ ^]^ The electrospun ATO‐supported Pt nanoparticles show an ORR activity that is almost the same as that of the cPt/C catalyst. The improved electroactivity and stability of the catalyst layer made Pt/ATO catalyst has great potential for PEMFC applications.^[^ ^79^ ^]^ When 1D ATO NFs employed as the support materials of the Pt catalyst for the MOR, the stability of Pt/ATO NF was effectively improved due to the corrosion resistance and porous fibrous structure of the ATO nanofiber.^[^ ^75^ ^]^

**FIGURE 11:**
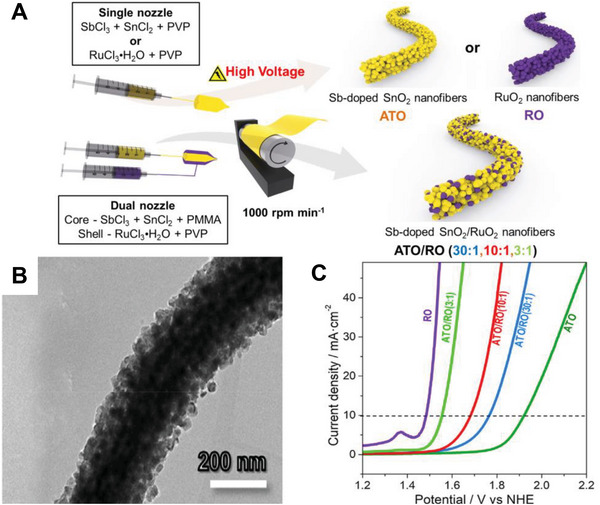
Preparation and electrocatalytic performance of Sb‐doped SnO_2_ (ATO)/RO NFs. A) The fabricating process of the ATO/RO composite, ATO, and RO nanofibers. B) TEM image of the ATO/RO(30:1) nanofibers. C) LSV curves of ATO, ATO/RO, and RO nanofiber electrodes in 1 m KOH solution (the scan rate is 10 mV s^−1^ and the voltage range is 0.0–2.5 V). Reproduced with permission.^[^ ^80^ ^]^ Copyright 2020, Elsevier.

The low oxygen evolution potential and high cost limited the wide utilization of RuO_2_ (RO)‐ and IrO_2_‐based anodes for electrochemical water treatment.^[^ ^80^ ^]^ For minimizing noble metal contents, Kim et al.^[^ ^80^ ^]^ prepared fibrous Sb‐doped SnO_2_ (ATO)/RO nanocomposite as anode materials (Figure 11). The 1D fibrous anodes are assembled of homogeneous ATO and RO NPs. The oxygen evolution potential of the materials could be successfully tuned by regulating the composition. The fibrous ATO/RO anodes demonstrate excellent electrocatalytic performance in oxidative organic degradation even when the RO content is only 3%. From this study, we believe that this strategy of designing composite nanofibers by using ATO as a medium can be extended to developing other efficient electrocatalytic and low‐cost anodes for high‐performance electrochemical water treatment.

In general, electrospun ATO nanofibers have excellent electrical conductivity and stability, even compared with carbon supports in acidic media.^[^ ^78^ ^]^ The improvement in electrospinning methods can further enhance the properties of ATO fiber itself. Through the electrospinning‐calcining‐grinding route, Ren et al. prepared short ATO nanofibers with good dispersion stability in water.^[^ ^81^ ^]^ Mudra et al.^[^ ^82^ ^]^ synthesized Fe^3+^‐doped and Fe_3_O_4_ NPs‐doped SnO_2_ nanofibers through a needle‐less electrospinning process and this needle‐less setup can generate abundant Taylor cones and provide high productivity. These methods have a certain reference value for improving the preparation of ATO fibers by electrospinning and using them as catalyst supports in the electrocatalytic application. With continuous research on the structure designing of electrospun fibers, more ATO nanofibrous supports with novel structures have also been developed. Lee et al.^[^ ^83^ ^]^ prepared Sb‐doped SnO_2_ NPs sandwiched between carbon nanofiber and carbon skin (CNF/ATO/C) via electrospinning and hydrothermal procedure. The CNF/ATO/C possesses a high specific capacity, superb cycling stability, outstanding high‐rate performance, and ultrafast cycling stability. On the other hand, the coaxial electrospinning approach is also used to prepare ATO nanofibers with advanced structures. Take TiO_2_@ATO nanofibers as an example,^[^ ^84^ ^]^ the continuous nanofibers show a dense structure and a smooth surface which consisted of a core of crystalline TiO_2_ and a shell of ATO NPs. The nanofibers calcined at 600°C displayed better electron conductivity. Thus, we have reason to believe that with the continuous improvement of electrospinning technology, 1D‐ATO nanofibers with excellent properties and advanced structures prepared via electrospinning technology will be better used in the fabrication of supported catalysts with enhanced electrocatalytic performance, stability, and low cost.

## 4 CONCLUSIONS AND PERSPECTIVES

**SCHEME 5:**
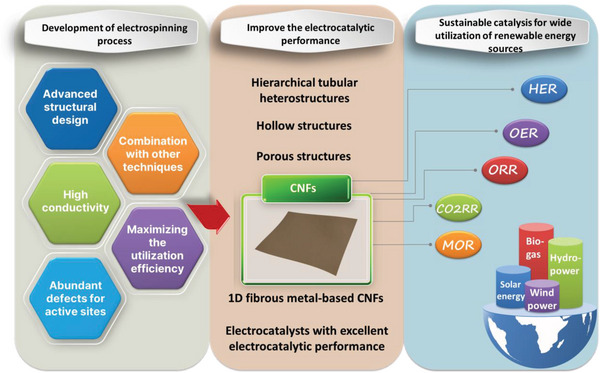
1D metal‐based CNF catalysts fabrication by electrospinning for sustainable catalysis in utilizing renewable energy sources.

Thanks to the synergy between highly dispersed active sites and particular substrates with the 1D nanostructure, fibrous carbon‐based nanoreactors have exhibited fascinating electrocatalytic performance in electrochemical reactions (Scheme 5). We have summarized the recent development of one‐dimensional fibrous carbon‐based catalysts from the electrospun fabrication process and characterization to the applications in catalyzing ORR, OER, and HER electrochemical reactions in this review. Particularly, the role of electrospinning technology in the fabrication of CNFs with advanced structures and excellent catalytic activity is extensively discussed. CNF‐based catalysts have made remarkable progress in optimizing active sites and electronic structures, however, many challenges remain in achieving high electrocatalytic performance in practical applications.

From the perspective of large‐scale industrial production, it is necessary to reduce the cost of the catalyst while ensuring the catalyst processes a high‐efficient catalytic performance in relatively long working hours.^[^ ^85^ ^]^ With regards to this, electrospun 1D fibrous carbon‐based nanoreactors could be deeply studied in the following aspects:

1. Enhancing the exposure of the active site by tailoring the micro/nanostructures of 1D CNFs can improve the catalytic performance of CNF‐based catalysts. Regulating the surface properties and structure of 1D CNF‐based catalysts by introducing heteroatoms can enrich the active sites on the catalytic surface. Furthermore, as a new frontier of heterogeneous catalysis, the active‐metal species of single‐atom catalysts (SACs) are atomically immobilized to the supports. This feature enables SACs to achieve 100% atomic utilization of active metals. Meanwhile, the coordination between those metal atoms and multiple heteroatoms can also produce synergistic effects for improving the catalytic performance of the catalysts.
2. In recent years, flexible and portable energy storage devices have rapidly developed. In this sense, electrospun CNFs are good candidates for flexible conductive networks along with the advantages of low cost and easy mass production.^[^ ^44f^ ^]^ Thus, it is of great scientific and practical significance to design and fabricate CNFs with superior ORR, OER, or HER electrocatalytic activity as high‐performance electrode materials. Recently, it was found that defect engineering is an effective strategy to improve catalytic performance by regulating the surface properties and electronic structure of catalysts. And due to the unique preparation technology, it is easy to introduce various defect sites in CNFs.^[^ ^86^ ^]^ Kim et al. reported the synthesis of Co and defect‐rich CNF as an efficient ORR electrocatalyst.^[^ ^86^ ^]^ Wang et al. developed the Co nanoparticles embedded in porous N‐doped CNFs that exhibit extraordinary OER and HER electrocatalytic performance.^[^ ^87^ ^]^ From these contributions, we have the confidence to believe that obtaining defect‐rich CNF‐based materials through exquisite design is a promising approach to improving the electrocatalytic property of novel catalysts for the utilization of flexible energy storage devices.
3. The electrospun support with excellent catalytic performance and electrochemical stability should be further explored, and the specific surface area and acid resistance of the supported non‐noble metals should be improved to prepare high‐activity and stable non‐noble metal HER or OER catalysts.
4. The combination of electrospinning technology with other processes (such as the ALD technology) for designing 1D nanoreactors with more advanced structures will be helpful to improve the stability and catalytic performance of CNF‐ catalysts. Moreover, combining the process design with theoretical calculations and molecular simulation techniques is useful in the research of chemical reaction paths, transition states, reaction mechanisms, and performance of CNF‐catalysts, and in investigating the actual macroscopic aspect from the electronic level of the catalytic mechanism. The combined use of these methods will provide a remarkably simple and powerful means for generating more efficient and stable carbon‐based catalysts.

Electrospinning is an interesting and promising technique to prepare carbon nanofibers, however, the process in many areas still requires further refinement and improvement. First, the variety of polymers used in electrospinning is limited and fiber preparation on a large scale with diameters below 100 nm (especially less than 10 nm) through the electrospinning process is still a major challenge. Second, the drum collector can acquire a large area of fibrous mesh. However, obtaining highly aligned fibers with substantial thickness and a relatively large area is still a tough issue despite several devices that have been designed to overcome this shortcoming. In this regard, the improvement of electrospinning devices is necessary for the expansion of its application fields. Third, the correlation between the structure of nanofibers and the processing parameters in the electrospinning process still needs to be systematically studied. Generally, the construction of fibrous materials with well‐defined arrays or hierarchical architectures is of great importance to fully achieve their potential in producing 1D fibrous carbon‐based catalysts with excellent catalytic performance. We have reason to believe that in the future, the electrospinning technique will become a powerful tool for producing 1D CNF catalysts with a broad range of functionalities and applications in energy storage and conversion.

## CONFLICT OF INTEREST STATEMENT

The authors declare no conflict of interest.
